# Novel Polymorphic Microsatellite Markers Reveal Genetic Differentiation between Two Sympatric Types of *Galaxea fascicularis*

**DOI:** 10.1371/journal.pone.0130176

**Published:** 2015-07-06

**Authors:** Yuichi Nakajima, Chuya Shinzato, Noriyuki Satoh, Satoshi Mitarai

**Affiliations:** 1 Marine Biophysics Unit, Okinawa Institute of Science and Technology Graduate University, Onna, Okinawa, Japan; 2 Marine Genomics Unit, Okinawa Institute of Science and Technology Graduate University, Onna, Okinawa, Japan; National University of Singapore, SINGAPORE

## Abstract

The reef-building, scleractinian coral, *Galaxea fascicularis*, is classified into soft and hard types, based on nematocyst morphology. This character is correlated with the length of the mitochondrial non-coding region (mt-Long: soft colony type, and nematocysts with wide capsules and long shafts; mt-Short: hard colony type, and nematocysts with thin capsules and short shafts). We isolated and characterized novel polymorphic microsatellite markers for *G*. *fascicularis* using next-generation sequencing. Based upon the mitochondrial non-coding region, 53 of the 97 colonies collected were mt-Long (mt-L) and 44 were mt-Short (mt-S). Among the 53 mt-L colonies, 27 loci were identified as amplifiable, polymorphic microsatellite loci, devoid of somatic mutations and free of scoring errors. Eleven of those 27 loci were also amplifiable and polymorphic in the 44 mt-S colonies; these 11 are cross-type microsatellite loci. The other 16 loci were considered useful only for mt-L colonies. These 27 loci identified 10 multilocus lineages (MLLs) among the 53 mt-L colonies (*N*_MLL_/*N* = 0.189), and the 11 cross-type loci identified 7 MLLs in 44 mt-S colonies (*N*_MLL_/*N* = 0.159). Significant genetic differentiation between the two types was detected based on the genetic differentiation index (*F*_ST_ = 0.080, *P* = 0.001). Bayesian clustering also indicated that these two types are genetically isolated. While nuclear microsatellite genotypes also showed genetic differentiation between mitochondrial types, the mechanism of divergence is not yet clear. These markers will be useful to estimate genetic diversity, differentiation, and connectivity among populations, and to understand evolutionary processes, including divergence of types in *G*. *fascicularis*.

## Introduction

Coral reef biodiversity estimates require unambiguous definitions of reef-building coral species because these constitute the structure of the reefs and they are increasingly endangered by both global and local disturbances. Polymorphic nuclear microsatellite loci provide invaluable information about complex sympatric species boundaries and about genetic differentiation and isolation. Next-generation sequencing is useful for surveying numerous genomic loci, including microsatellite markers. Shinzato et al. [1] sequenced the entire genome of the coral, *Acropora digitifera*, but genomic data for most other reef-building corals is presently lacking.

Reef-building, scleractinian corals of the genus *Galaxea* are distributed throughout the Indo-western Pacific. *Galaxea* comprises seven species, four of which (*Galaxea astreata*, *G*. *fascicularis*, *G*. *longisepta*, and *G*. *paucisepta*) occur around Okinawa Island [2]. *Galaxea fascicularis* (Linnaeus, 1767) cannot be mistaken for any other species. It is widely distributed [2], and is one of the dominant coral species in reefs around Okinawa, Japan. It is a gonochoric, broadcast spawning species; female colonies produce egg bundles, and male colonies form bundles consisting of sperm and infertile pseudo-eggs [3–5]. In Okinawa these colonies are classified into soft and hard types based on nematocyst (microbasic p-mastigophore) morphology [6]. These features are also correlated with the length of the non-coding region between the mitochondrial genes, cytochrome b (*cyt b*) and NADH dehydrogenase subunit 2 (*nad 2*) (mt-Long: soft colony type and nematocysts with wide capsules and long shafts; mt-Short: hard colony type and nematocysts with thin capsules and short shafts) [7,8]; therefore, these two types represent novel clades or species. Interestingly, gamete fertilization experiments revealed that these two types show partial reproductive isolation [9]. However, the reproductive barriers that separate these sympatric types and that cause their genetic differentiation are not obvious. *Galaxea fascicularis* has several distinctive color morphs (B: entire polyp is pale brown, BG: brown with greenish oral disks, Gs: brown, but with tentacles on the major septa that are light green, Gt: brown, but with lateral tentacles that are light green, Wt: brown with pale green (almost white) fluorescent lateral tentacles, and Ft: brown polyps with green fluorescent lateral tentacles) [8,10]. However, these color morphs are entirely unrelated to mitochondrial type [8,11].

For *G*. *fascicularis*, Suzuki et al. (unpublished data) developed five microsatellite markers (GenBank accession number: AB272099-AB272103), and Chen et al. [12] attempted to characterize the markers using colonies from two Chinese populations. However, two of these loci showed significant deviation from Hardy-Weinberg equilibrium (HWE) in both populations. Abe et al. suggested that in a third microsatellite locus (locus MS-1), the genotypic pattern is related to the length of the non-coding region [8], and that hybrid larvae show allelic features of both types [9]. However, locus MS-1 (AB272101) was identified as a zooxanthellan locus by Chen et al. [12]. Therefore, genetic conclusions drawn from it were misleading.

Acquisition of additional microsatellite and other genetic markers is essential for definitive population genetics studies in order to examine genetic differentiation, hybridization, and boundaries between types within species. We isolated and characterized novel polymorphic microsatellite markers for *G*. *fascicularis* using next-generation sequencing. We characterized one population based on the ratio of mitochondrial types and investigated clonality of each mitochondrial type and genetic differentiation between mitochondrial types within that population.

## Methods

### Ethical statement

All samples for this study were collected in strict accordance with good animal practice as defined by the relevant national and/or local animal welfare bodies, and for sample collection was approved by the Okinawa Prefectural Governor, Hirokazu Nakaima (No. 25–53).

### Isolation of microsatellite and primer design

We constructed a genomic DNA library using sperm of a *Galaxea fascicularis* mt-L colony (mitochondrial type was determined following sequencing). Genomic DNA was isolated using proteinase K and phenol-chloroform extraction and purification with ethanol precipitation. Extracted DNA was sequenced using 250 bp paired end reads on a MiSeq sequencer (Illumina) according to manufacturer’s instructions. Sequencing was performed with samples of other organisms using different index adaptors in a single MiSeq run. We performed subsequent bioinformatics analyses using default software parameters unless otherwise indicated. We did not use a quality filter for raw sequence data. Read pairs of at least 100 bp in both sequence directions were retained using SolexaQA [13]. Duplicated sequences were removed with filterPCRdupl ver. 1.01 and sequencing adapters were trimmed with fastq-mcf in ea-utils ver. 1.1.2–537 [14]. Sequences of each read pairs were merged. Then assembled sequences longer than 100 bp were selected. Detection of simple sequence repeats and PCR primer design in each assembled sequence were performed with PAL_FINDER ver. 0.02.04 [15]. In order to select microsatellite loci that might be highly variable, we selected primer pairs amplifying longer repeat stretches (thresholds: 3 mer repeats are 10 or more and 4 mer are eight or more, respectively).

### Genotyping by PCR and fragment analysis

**Fig 1:**
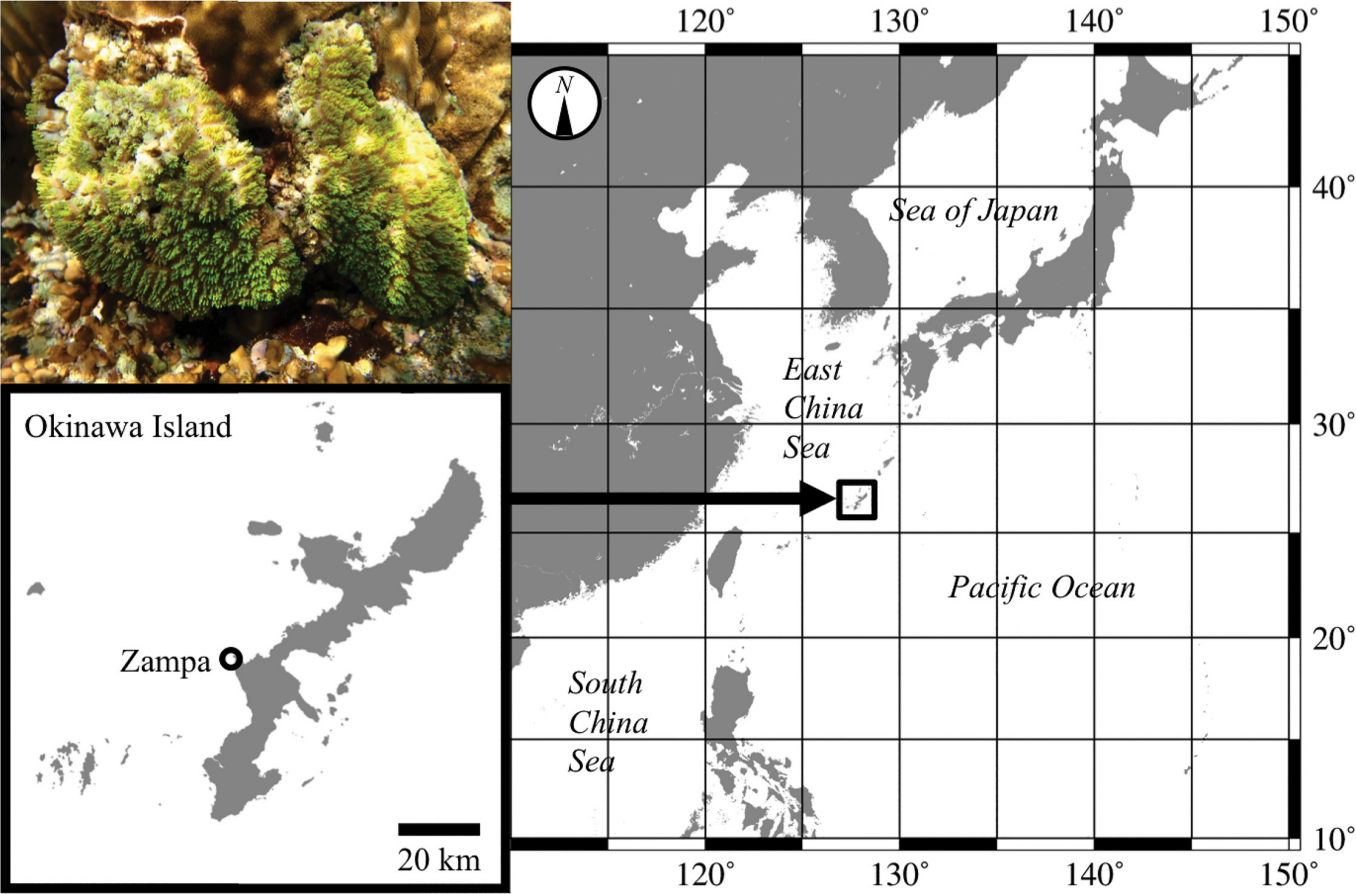
Photograph (upper left) and map showing the sampling location for *Galaxea fascicularis*. Zampa is located on Okinawa Island, Japan.

To characterize microsatellite loci, we screened 97 colonies of *G*. *fascicularis* collected randomly at Zampa, Okinawa Island (26°26′20″N/127°42′40″E) (Fig 1). Color morphs were ignored in this study, because they are not correlated with mitochondrial type [8, 11]. Samples were preserved in ethanol and brought to the laboratory, where genomic DNA was extracted using a DNeasy Blood & Tissue kit (Qiagen). To analyze polymorphism and amplification of designed primer sets and to identify mitochondrial types, we used the tailed primer method to perform PCR. The reaction mixture (5 μL) contained template DNA (< 100 ng), AmpliTaq Gold 360 Master Mix (Qiagen), and three primers for each locus: a non-tailed forward primer (0.2 μM), a reverse primer with a U19 sequence tail (0.2 μM), and a U19 (5’-GGTTTTCCCAGTCACGACG-3’) primer (0.5 μM) fluorescently labeled with FAM, VIC, NED, or PET, based on the method by Schuelke [16]. Furthermore, 188–1 (5’- GAATAGGGTATACTAGCAGGTC -3’, see [7]), 188-R3-U19 (5’- GGTTTTCCCAGTCACGACGCATCATTATCCTCTTCAAGG -3’), and U19 primer fluorescently labeled with FAM were used for identification of mitochondrial types (mt-L or mt-S) based upon the non-coding region between *cyt b* and *nad 2*. Amplifications were carried out under the following conditions: 95°C for 9 min; followed by 35 cycles at 95°C for 30 s, 54°C for 30 s, and 72°C for 1 min; and a final extension at 72°C for 5 min. Amplified PCR products with an added internal size standard (GeneScan 600 LIZ; Applied Biosystems) were analyzed using an automated, capillary-based DNA sequencer, ABI 3130xl Genetic Analyzer (Applied Biosystems), and GeneMapper ver. 3.7 (Applied Biosystems). Loci showing non-amplification or multiple non-specific peaks were excluded from further analysis. Furthermore, loci with little polymorphism or few heterozygotes (e.g., almost all colonies were homozygous) were also excluded.

### Statistical analysis

Multilocus lineages (MLLs) were employed in order to avoid genotyping samples incorrectly (see [17]). In this study, if only one locus differed in genotype, while all other successful loci coincided, that colony was considered to be clonal, derived asexually by fragmentation. Either a somatic mutation or a scoring error was assumed to be responsible for the variable locus. When we found a somatic mutation or a scoring error while genotyping mt-L colonies, the locus was deleted from the list of retained loci. If similar problems were encountered in an mt-S locus after characterization in mt-L, that allele was removed altogether from the analysis (input as 0 to GenAlEx allele data).

For successful microsatellite loci, the number of MLLs was counted in order to estimate clonality using GenAlEx ver. 6.5 [18]. Clonality was estimated with the following index: *N*_MLL_/*N* (*N*_MLL_: the number of MLLs, *N*: the number of colonies). After removal of clonal replicates from the data set, the number of alleles (*N*_A_), values of expected and observed heterozygosity (*H*_E_ and *H*_O_, respectively), and deviation index (*F*_IS_) from HWE for each type were also evaluated with GenAlEx. Using all retained loci, linkage disequilibrium was estimated for mt-L and mt-S types using Genepop ver. 4.2 (available at http://genepop.curtin.edu.au/) [19,20] with default conditions. The significance level was adjusted using a false discovery rate (FDR) correction at the 95% confidence level [21].

We calculated the *F*_ST_ value as a genetic differentiation index between types using GenAlEx. Population structure between the two types based on Bayesian clustering was inferred using STRUCTURE ver. 2.3.4 [22]. A burn-in period of 100,000 followed by 1,000,000 Markov chain Monte Carlo (MCMC) replications was used for population clustering without prior information under the admixture model and assuming independent allele frequencies between clusters [23]. In the admixture model, individuals were assumed to be drawn purely from genetic clusters (the number of assumed genetic clusters is shown as *K*) and were allowed to have mixed ancestry [22,23]. The simulations included 10 iterations, and the number of assumed genetic clusters was one to six. Mean alpha value and range were confirmed for each *K* to ensure convergence of MCMC replications, following instructions for STRUCTURE. After calculation of the mean value of log probability, Ln P(D), the model choice criterion to detect the most probable value for each *K* [22], detection of the number of *K* clusters that best fit the data was determined using the method of Evanno et al. [24], as implemented in STRUCTURE HARVESTER [25]. ∆*K* is an ad hoc quantity for predicting the number of possible clusters [24]. Run data were merged with CLUMPP ver. 1.1.2b [26]. Furthermore, discriminant analysis of principal components (DAPC) was conducted in R ver. 3.0.2 [27] using the package adegenet ver. 1.3–9.2 [28], to represent genetic patterns for each MLL. This clustering method does not assume a particular model, so it is free of assumptions about HWE and linkage equilibrium [28]. To avoid unstable output, we set ≤one-third of the MLLs as the number of principal components to retain (PCs: 5; ≤one-third of 17 MLLs, see Results and Discussion).

## Results and Discussion

### Determination of mitochondrial type

From the non-coding region between *cyt b* and *nad 2*, 53 colonies from Zampa were identified as mt-L while 44 were categorized as mt-S. No ambiguous or unexpected mitochondrial types were detected in this study. The finding that the number of mt-L colonies exceeded the number of mt-S colonies mirrors the results of Abe et al. [9] (mt-L: 22, mt-S: 13), but not those of Watanabe et al. [7] (mt-L: 1, mt-S: 20). The ratio of types differs in every population [7]. These two types are clearly sympatric at Zampa; however, further study is needed to clarify their distributions. Though this species also inhabits the temperate zone near mainland Japan [2,29], it is possible that other types are also present there.

### Isolation of microsatellite markers

We obtained 1,730,565,720 bp (3,507,101 read pairs) of raw sequence data and each read pair was assembled. We used 3,368,983 assembled sequences longer than 100 bp (1,155,663,709 bp, average 343 bp) for SSR identification using PAL_FINDER. These SSRs were used for microsatellite detection. One hundred twenty primer pairs were selected from which to design microsatellite primer pairs (3 mer repeats: 60 loci, 4 mer: 60). With these 120 pairs, 27 loci were successfully amplified from 53 colonies of the mt-L type, without somatic mutations or scoring errors, using the 53 mt-L colonies. Eleven of the 27 loci were identified as cross-amplifiable, polymorphic microsatellite markers (Table 1); somatic mutations or scoring errors were found in mt-S colonies for five loci. Sixteen of the 27 loci were characterized as useful only for mt-L colonies (Table 2). Four loci, Gfas3_011 and Gfas3_020 (effective for both mt-L and mt-S) and Gfas3_017 and Gfas_037 (effective for only mt-L), possess imperfect repeats within the repeat. These loci may not have followed a simple, stepwise mutation process, although they are likely to fit the infinite allele model [30].

**Table 1:** Characteristics of the 11 polymorphic, cross-type microsatellite loci for mt-L and mt-S in *Galaxea fascicularis*.

Locus	Repeat motif	Primer sequence (5'-3')		Size range (bp)	*N*_A_	*H*_E_	*H*_O_	*F*_IS_	GenBank accession No.
Gfas3_011	(AGC)_4_(AGT)_39_GGTAGAAGTGGTAGCAGT(ATT)_2_ATC(AGT)_7_	F: CATACTAGCAGCGGCATACG	L:	242–471	14	0.905	0.700	0.227	LC030476
		R: U19-AACAACCATGTGCTCGCC	S:	242–260	6	0.633	0.714	-0.129	
Gfas3_020	(TAC)_33_(TGC)_2_(TAC)_2_TAT(TAC)_3_TAA(CAA)_2_TGATAATGACGACAA(TGA)_6_	F: U19-CCACTGACAAATCACG	L:	176–302	9	0.785	0.800	-0.019	LC030477
		R: CTCAACTAGAGGCAAGAGCG	S:	223–275	9	0.875	0.833	0.048	
Gfas3_045	(AAT)_10_AGTAATAAC(AAT)_2_	F: AAGGCGCTGTAATCGACG	L:	178–230	7	0.845	0.800	0.053	LC030478
		R: U19-AGGGGACCTTGATTTGCC	S:	159–224	9	0.857	1.000	-0.167	
Gfas3_059	(TTA)_12_	F: TTGCCGAGTGGTTTAGGG	L:	249–460	11	0.890	0.800	0.101	LC030479
		R: U19-AGTCATTCAGACCAGCTTGC	S:	271–350	10	0.888	1.000	-0.126	
Gfas3_060	(TTA)_12_	F: CATGTTCAATCACGCAGC	L:	174–209	10	0.890	1.000	-0.124	LC030480
		R: U19-TCAGTCTTAAGATCATCGCC	S:	164–185	6	0.796	0.429	0.462	
Gfas4_067	(TATC)_30_	F: ACGCAATTCAGCTCTCCG	L:	209–318	11	0.825	0.700	0.152	LC030481
		R: U19-TTGGACGTTTAGGCCACC	S:	185–272	7	0.840	1.000	-0.190	
Gfas4_079	(TTAC)_2_CTAC(TTAC)_25_	F: AACATTTGATTCCCACTCGG	L:	211–291	12	0.900	1.000	-0.111	LC030482
		R: U19-CAAGAGTGTCGGGCAACG	S:	203–259	4	0.582	0.429	0.263	
Gfas4_081	(GATT)_26_	F: ACACACGGCATTCATGG	L:	287–357	11	0.875	0.600	0.314	LC030483
		R: U19-TTTTGTGAAAACGAAAATGG	S:	268–343	8	0.827	0.714	0.136	
Gfas4_090	(TAGA)_25_	F: TTAGCCTCTCCACTTTACGG	L:	188–232	8	0.825	0.800	0.030	LC030484
		R: U19-TTTCTGGCCATCTGCG	S:	155–245	8	0.778	0.833	-0.071	
Gfas4_091	(AGAT)_25_	F: U19-CGCTAGTGAAGACCAGCC	L:	144–229	10	0.860	1.000	-0.163	LC030485
		R: AGCCTTTGGCTGTGATGC	S:	140–233	8	0.847	0.667	0.213	
Gfas4_110	(TGGT)_10_(TGAT)_10_	F: TGGCAGGGTGTTCTGC	L:	276–329	10	0.855	0.900	-0.053	LC030486
		R: U19-CAGCAGAAATTTCCTCTTCC	S:	275–428	9	0.875	0.500	0.429	

The size range of amplification products includes the U19 sequence.

*N*_A_: number of alleles per locus, *H*_E_: expected heterozygosity, *H*_O_: observed heterozygosity, *F*_IS_: deviation index from HWE.

These loci did not deviate from HWE.

**Table 2:** Characteristics of the 16 polymorphic microsatellite loci for mt-L in *Galaxea fascicularis* (not successfully amplified in mt-S).

Locus	Repeat motif	Primer sequence (5'-3')	Size range (bp)	*N*_A_	*H*_E_	*H*_O_	*F*_IS_	GenBank accession No.
Gfas3_016	(AAG)_34_	F: CGAGCCTGCATTATCGG	278–373	10	0.820	0.800	0.024	LC030487
		R: U19-GTATATGGGTGGAGCG						
Gfas3_017	(AGT)_3_AGC(AGT)_34_AGG(AGT)_24_GGTAGT(AGC)_4_	F: CTTTTAGCCCGCTTGACC	173–298	12	0.880	0.700	0.205	LC030488
		R: U19-TGCTACTACCTGACTGCTGC						
Gfas3_028	(GTA)_30_	F: TTGTTGGCTAACACCCTCG	200–266	10	0.870	0.700	0.195	LC030489
		R: U19-TATGCCTCCTGCCACTCC						
Gfas3_032	(AAG)_30_	F: U19-TTGTGCGCTAGGATCGG	237–319	11	0.885	0.600	0.322	LC030490
		R: TTCCCCTGTTTTACTTTGCC						
Gfas3_037	(TAG)_2_(TGG)_3_(TAG)_28_TGG(TAG)_4_TGGTAA(TAG)_2_TGG(TAG)_3_(TGG)_3_	F: GCAATGATGGAGAGAAGGG	189–244	7	0.610	0.500	0.180	LC030491
		R: U19-CCTCTGCCACTACCACC						
Gfas3_049	(TTA)_10_	F: AATCGGATCAGAGCGTGG	199–223	8	0.800	0.900	-0.125	LC030492
		R: U19-TCATTGCGCCTTTCTTCC						
Gfas3_050	(ATT)_10_	F: AATTAGATAGATGCCGTGCC	271–374	10	0.820	0.500	0.390*	LC030493
		R: U19-TTTGGGCCAGCTTAGACC						
Gfas3_053	(TAA)_12_	F: CCTGGAAACAATGAAGGGC	190–215	6	0.795	0.800	-0.006	LC030494
		R: U19-CCCAAGAGAAGTCAGCC						
Gfas4_061	(TATG)_35_	F: AGGCAGGTCCACATCAGG	170–301	10	0.860	1.000	-0.163	LC030495
		R: U19-TTTATGGAAACCACGAGAGC						
Gfas4_074	(TAAG)_27_	F: TTTGGAGGAAGCCTGTGG	177–291	10	0.845	1.000	-0.183	LC030496
		R: U19-TTTCCTCTTCAAAAGGCCC						
Gfas4_076	(CTAA)_26_	F: U19-CCATCCATTGTATATGCC	319–391	7	0.815	0.300	0.632**	LC030497
		R: CACCTTTTCTCGAGGATACC						
Gfas4_083	(TACA)_26_	F: ACATGAAGGGAGGGAGCC	199–293	10	0.865	0.800	0.075	LC030498
		R: U19-ATGCACGACCCCATAAGC						
Gfas4_084	(TCTA)_26_	F: TCCTAGTGTTGGCAGGGC	132–229	11	0.800	0.800	0.000	LC030499
		R: U19-AACAGATGCACCGCAGG						
Gfas4_106	(GTCA)_3_GTCG(GTCA)_10_	F: CAATTCTGTGAGAAAGGCG	271–321	6	0.760	0.500	0.342	LC030500
		R: U19-AAAATTTGACAGTGTCTGGC						
Gfas4_107	(GACT)_2_AACTGACG(GACT)_10_	F: AACATCTCCGCGAAGGC	185–229	6	0.650	0.600	0.077	LC030501
		R: U19-AGGAACCGGGAAGTTTGG						
Gfas4_114	(AGAC)_2_AAAG(AGAC)_8_	F: CATTTTAACGGGAACCGC	114–155	8	0.775	0.600	0.226	LC030502
		R: U19-TTTTGCACGATCCCACG						

The size range of amplification products includes the U19 sequence.

*N*_A_: number of alleles per locus, *H*_E_: expected heterozygosity, *H*_O_: observed heterozygosity, *F*_IS_: deviation index from HWE.

*Significant deviation from HWE (*P < 0.05, **P < 0.01).

### Clonality by multilocus lineage for each type

These 27 loci indicated 10 MLLs in 53 mt-L colonies (*N*_MLL_/*N* = 0.189), and 11 cross-type loci identified 7 MLLs in 44 mt-S colonies (*N*_MLL_/*N* = 0.159). This shows that there is little difference in the degree of clonality between types. In some coral species, clonality depends on location [31,32]. High storm frequencies tend to cause higher levels of clonality in populations of *Pocillopora verrucosa* [33] due to fragmentation. Thus, clonality appears to be less influenced by type than by environmental or geographic factors. Further collection of samples at additional locations will help to confirm this observation, and spatial correlations based on locality data will be needed to estimate kinship among colonies.

Clonal reproduction likely enhances the probability of population persistence when environmental conditions are unsuitable for sexual reproduction, or when low population densities supply fewer mating opportunities (i.e. Allee effects) [34,35]. If clonal colonies are dominant in a population, the success rate of fertilization appears to decrease considerably. In most cases, under experimental conditions, self-fertilization by sexual reproduction rarely occurs [36], and gametes derived from colonies with high kinship, tend to fail to fertilize in some hermaphroditic corals, such as some *Acropora* species [37]. However, the hermaphroditic coral genus, *Goniastrea*, especially *Goniastrea favulus*, shows a very high rate of self-fertilization compared to *Acropora* and *Montipora* [38,39]. While it is not clear whether higher kinship affects fertilization in gonochoric species, in *Galaxea fascicularis*, higher kinship may prevent sexual reproduction. Furthermore, populations with decreased clonal diversity are more vulnerable to pathogens, which may be increasing due to increases in sea-surface temperature [32,40]. Nevertheless, local adaptation may also decrease mortality due to pathogens, with persistence of the most fit genotypes [41]. Thus adaptation may decrease the variety of genotypes due to local selection; however, it may prevent local extinction as long as catastrophic environmental changes do not occur.

### Characterization of microsatellite markers

The isolated markers revealed 6–14 alleles per locus for 10 MLLs in mt-L and 4–10 for 7 MLLs in mt-S. Fifteen of 182 alleles in 11 loci were shared between mt-L and mt-S types. Some common alleles may accord by chance due to size homoplasy of the microsatellite region [42]. *H*_E_ and *H*_O_ for the 27 mt-L loci ranged from 0.610 to 0.905 and from 0.300 to 1.000, respectively. *H*_E_ and *H*_O_ of mt-S in 11 loci ranged from 0.582 to 0.888 and from 0.429 to 1.000, respectively. Most of these loci did not show significant deviation from HWE, except for Gfas3_50 and Gfas4_076 in mt-L (Table 2). There is no HWE deviation in 11 loci in mt-S. In addition, there is no significant linkage disequilibrium for all combinations of loci among either mt-L (27 loci) or mt-S (11 loci).

### Genetic differentiation between mt-L and mt-S colonies

**Fig 2:**
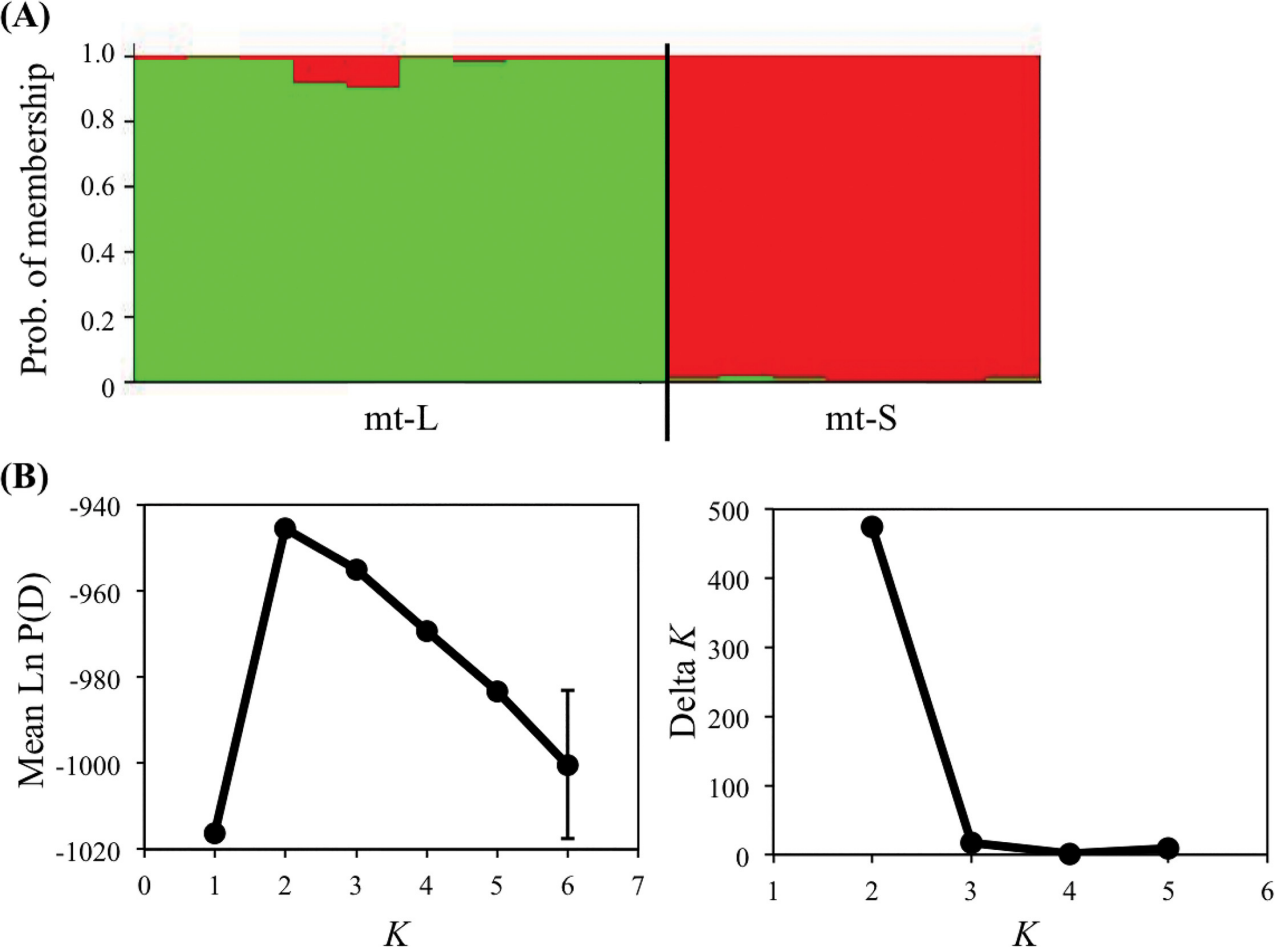
Genetic differentiation between mt-L and mt-S colonies by analysis with STRUCTURE. (A) STRUCTURE bar plot as a result of a Bayesian clustering analysis for 17 MLLs (mt-L: 10; mt-S: 7) of *Galaxea fascicularis* at Zampa, genotyped using 11 cross-type microsatellite loci. The probability of membership in each MLL is shown as a vertical bar. (B) Graphs of mean log probability (Ln P(D), the model criterion of choice to detect the most probable *K* [22]) values (*K* = 1 to 6) across 10 iterations per *K* (the assumed number of populations), and Δ*K* values based on the rate of change in Ln P(D) between successive *K* values (*K* = 2 to 5) for detecting the number of *K* clusters that best fit the data suggested by Evanno et al. [24].

**Fig 3:**
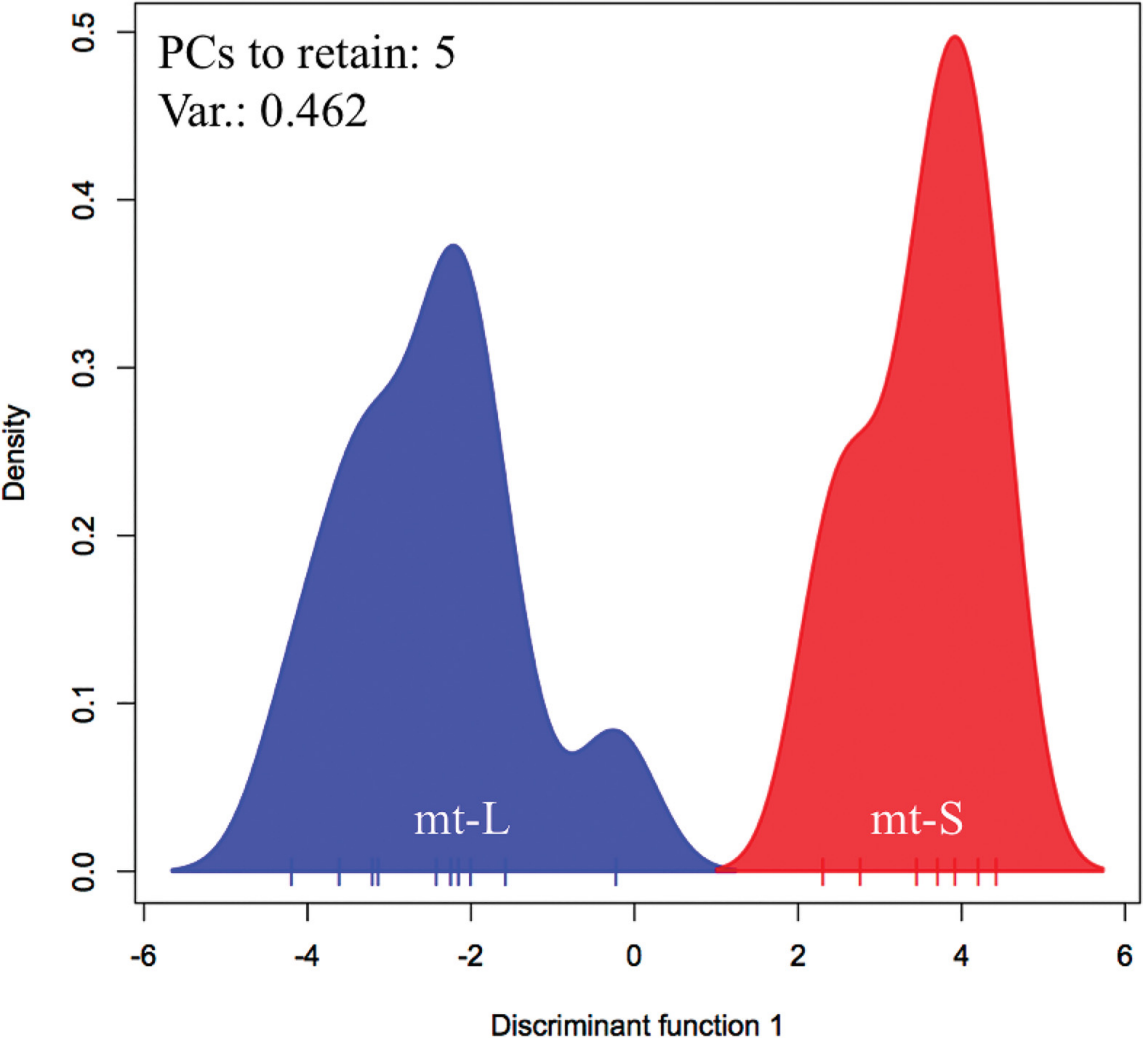
Genetic differentiation between mt-L and mt-S colonies by discriminant analysis of principal components (DAPC). Five PCs were retained.

Significant genetic differentiation between types was detected based on the genetic differentiation index (*F*_ST_ = 0.076, *P* = 0.001). Furthermore, the results of Bayesian clustering using STRUCTURE also indicated that these two types are genetically isolated (Fig 2). When the number of genetic clusters was assumed to be two (*K* = 2), ∆*K* as ad hoc quantity for predicting of *K* and mean Ln P(D) as posterior probability of the data given *K*, suggested the largest value (∆*K* = 475.17, mean Ln P(D) = -945.52), which were indicators of most probable *K* value. DAPC also indicated obvious differentiation between the mt-L and mt-S clusters (Fig 3). This result showed that the most probable number of genetically clustered populations was two. It is also evident that there is genetic differentiation at the population level between these two sympatric types; therefore they appear to represent legitimate species. These markers provide evidence of significant differentiation between mt-L and mt-S; however, they may underestimate differentiation of the two *G*. *fascicularis* types, because the 11 cross-type loci are relatively well conserved between types.

Species boundaries of other coral genera are often morphologically ambiguous. For example, in *Acropora*, hybridization has been confirmed both in the field [43,44] and in the laboratory [36]. Moreover, in that genus, morphological classification conflicts with genetic phylogeny [45] and there are cryptic species [46,47]. Furthermore, in the family Pocilloporidae, the definition of species has been debated due to the incongruence of phylogenetic data with morphotypes (*Pocillopora*: [48,49]; *Seriatopora*: [50]; *Stylophora*: [51]). The only species in the family Helioporidae, *Heliopora coerulea*, is genetically divided into two clades that are related in terms of shape (small-branch or flat type) [52]. In some species, including marine invertebrates, related species have arisen through sympatric speciation [53]. The two types of *G*. *fascicularis* may have occurred by sympatric divergence; however, the mechanism has not identified. Therefore, more detailed ecological distribution patterns of both types and genetic differentiation need to be examined. In general, classification of reef-building corals will need to be based upon both morphology and genetics in order to estimate species diversity and other biological parameters.

### Future applications

This study reported 27 novel microsatellite markers that are useful for mt-L of *G*. *fascicularis*. Eleven of these can be used for both mt-L and mt-S as cross-type markers. Genotypic data obtained in this study revealed definite genetic differentiation between mt-L and mt-S, suggesting partial reproductive isolation. A recent study suggested that skeletal and color morphs of *G*. *fascicularis* appear related to such environmental factors as salinity and light intensity, rather than to genetic factors, such as mitochondrial type [11]. The microsatellite markers developed in this study may provide further information about the relationship between morphs and nuclear genotypes, and may elucidate evolutionary processes in the genus *Galaxea*. They will be available for population genetics studies of *G*. *fascicularis*, and they will be necessary in order to estimate genetic diversity, differentiation, connectivity among populations, and evolutionary processes, including divergence of types in *G*. *fascicularis*.
